# Different profiles of body mass index variation among patients with multidrug-resistant tuberculosis: a retrospective cohort study

**DOI:** 10.1186/s12879-020-05028-0

**Published:** 2020-04-28

**Authors:** Alhassane Diallo, Boubacar Djelo Diallo, Lansana Mady Camara, Lucrèce Ahouéfa Nadège Kounoudji, Boubacar Bah, Fulgence N’Zabintawali, Miguel Carlos-Bolumbu, Mamadou Hassimiou Diallo, Oumou Younoussa Sow

**Affiliations:** 1 INSERM, U1137, CIC-EC 1425, Department of Epidemiology, Biostatistics, and Clinical Research, AP-HP, Hospital Bichat, University Paris Diderot, Paris, France; 2 Faculté des Sciences et Techniques de la Santé, Université Gamal Abdel Nasser de Conakry, Service de Pneumo-Phtisiologie, CHU Conakry, Hôpital National Ignace Deen de Conakry, Conakry, Guinea; 3 Centre Antituberculeux de la Carrière, Conakry, Guinea; 4 Laboratoire National des Mycobactéries, Conakry, Guinea; 5 Urgences réanimation centre hospitalier Sud Essonnes CHSE, Paris, France; 6 Centre population et développement, Institut de recherche pour le développement, Université Paris Descartes, Paris, France

**Keywords:** BMI, Multidrug-resistant, Tuberculosis, Latent mixed models

## Abstract

**Background:**

Despite the predictive role of body weight variation in treatment outcome in multidrug-resistant tuberculosis (MDR-TB), few corroborating data are available. We studied weight variation in patients with MDR-TB to identify groups of weight change and to determine factors that influence these changes.

**Methods:**

We analyzed patients with rifampicin resistance who were treated with an MDR-TB treatment regimen between June 07, 2016 and June 22, 2018 at three major drug-resistant TB centers in Guinea. Patients were seen monthly until the end of treatment. Clinical outcome was the body mass index (BMI). We used a linear mixed model to analyze trajectories of BMI and a latent class mixed model to identify groups of BMI trajectories.

**Results:**

Of 232 patients treated for MDR-TB during the study period, 165 were analyzed. These patients had a total of 1387 visits, with a median of 5 visits (interquartile range, 3–8 visits). Monthly BMI increase was 0.24 (SE 0.02) per kg/m^2^. Factors associated with faster BMI progression were success of MDR-TB treatment (0.24 [SE 0.09] per kg/m^2^; *p* = 0.0205) and absence of lung cavities on X-ray (0.18 [0.06] per kg/m^2^; *p* = 0.0068). Two groups of BMI change were identified: rapid BMI increase (*n* = 121; 85%) and slow BMI increase (*n* = 22; 15%). Patients in the slow BMI increase group were mostly female (68%) had no history of TB treatment (41%), had a positive HIV infection (59%), and had a more severe clinical condition at baseline, characterized by a higher frequency of symptoms including depression (18%), dyspnea (68%), poor adherence to MDR-TB treatment (64%), lower platelet count, and higher SGOT. These patients also had a longer time to initial culture conversion (log-rank test: *p* = 0.0218).

**Conclusion:**

Quantitative BMI data on patients with MDR-TB treated with a short regimen allowed the identification of subgroups of patients with different trajectories of BMI and emphasized the usefulness of BMI as a biomarker for the monitoring of MDR-TB treatment outcome.

## Background

Multidrug-resistant tuberculosis (MDR-TB) is defined as resistance to the two powerful anti-TB drugs isoniazid (INH) and rifampicin (R). In 2016, the World Health Organization (WHO) reported 600,000 new cases of MDR-TB globally [1] with 136 cases in Guinea (Conakry) [2].

In a context of limited resources like Guinea, identification of a biomarker of MDR-TB treatment response that is easily measured and accessible in clinical practice would be beneficial for the management of patients and for tuberculosis control programs. Numerous studies have shown that malnutrition measured by body weight is associated with a poor MDR-TB treatment outcome, probably due to a complex relationship between lessened energy demands and decreased nutritional intake, and suggest that weight loss is a potential biomarker of treatment response [3–7]. Moreover, a longitudinal study showed that weight variation during the first 6 months differed according to the treatment outcome, a poor outcome being associated with decreasing weight over time [7]. The underlying hypothesis of the mixed linear model used to analyze weight change in this study is that all patients have a unique mean profile of weight trajectory over time. But this assumption of homogeneous weight change seems untenable, because patients with MDR-TB often differed in the severity of the disease at baseline in terms of clinical presentation, number of episodes of TB, presence of HIV co-infection or comorbidity (diabetes), susceptibility to drug toxicity and resistance. Hence the need to identify homogeneous groups of patients with different weight changes, using an appropriate methodology as latent class models, and to characterize them, which would enable identification of at-risk patients thereby ensuring more efficient use of resources and optimal patient management.

To the best of our knowledge, no study has reported the identification of weight change groups in patients with MDR-TB. Here, we report data from a retrospective cohort study of patients treated with a standardized 9-month treatment regimen in Guinea. We aimed to (a) specify weight over time as well as factors that influence it, (b) identify different groups of weight change, and (c) determine the factors associated with these groups.

## Methods

### Study design and population

**Fig. 1:**
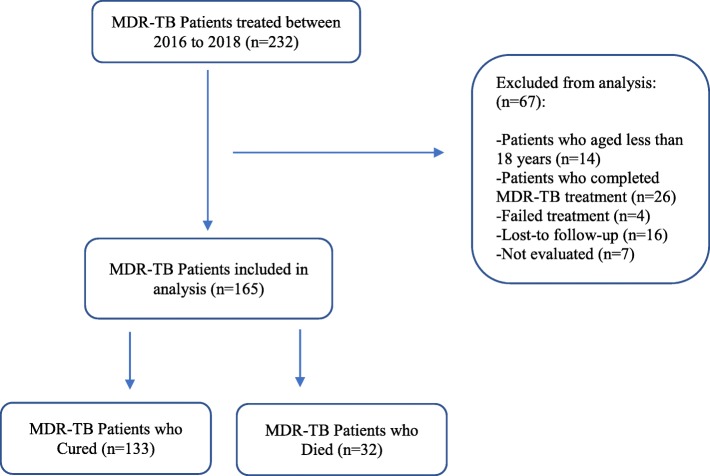
Flowchart of MDR-TB patients’ selection in the study

We conducted a retrospective, multicenter, longitudinal, cohort study at three referral drug-resistance TB centers in Guinea (Hospital Center University of Ignace-Deen, Carrière and Tombolia). We analyzed patients with rifampicin (RIF) resistance who received an MDR-TB treatment regimen between June 07, 2016 and June 22, 2018 and who died for any reason or were cured according to the 2013 WHO recommendation [8] (Fig. 1). We assumed that all RIF resistant strains are MDR, and in Guinea, as recommended by WHO, any patients with resistance to RIF was treated as MDR-TB for patients with high resistance; the test was repeated for patients at low risk of resistance. Drug resistance was diagnosed using the Xpert MTB/RIF test. According to the guideline for MDR-TB management in Guinea [2], patients naive to second-line anti-TB drugs were treated with a 9-month regimen consisting of an intensive phase lasting a minimum of 4 months including moxifloxacin, kanamycin, clofazimine, prothionamide, pyrazinamide, ethambutol, and INH at high dose. The intensive phase was then followed for 5 months by the continuation phase consisting of administration of four drugs: moxifloxacin, clofazimine, pyrazinamide, and ethambutol. Patients were seen at baseline and followed up at monthly visit for 9 months. The study was approved by the National Ethics Committee for Heath Research (NECHR) attached to the Ministry of Health (Conakry, Guinea).

### Outcome and predictive variables

Clinical outcome was the body mass index (BMI) calculated using the formula [weight/(height)^2^]. Weight, sputum smear, and sputum culture were obtained monthly during MDR-TB treatment. Sputum smear is a direct microbiological examination performed using the Ziehl-Neelsen method and sputum culture is based on solid culture according to the Lowenstein-Jensen method. Sputum smear conversion was defined as two consecutive negative sputum smears taken at least 30 days apart following an initial positive sputum smear. Similarly, culture conversion was defined as two consecutive negative cultures taken at least 30 days apart following an initial positive sputum culture. Time to initial sputum smear (or culture) conversion was defined as the time in months from the date of start of MDR-TB treatment to the date of specimen collection for the first of two consecutive negative sputum smear (or culture) results, even if a subsequent positive sputum smear (or culture) result occurred later. Demographic and clinical data included age, gender, residence, HIV infection status, history of the previously treated TB, the presence of cavities on chest X-ray determined by the senior radiologist, baseline data on weight, clinical symptoms (as chest pain or cough), and laboratory data (creatinine, SGOT and SGPT, leukocyte count, hemoglobin, and neutrophil count). Additionally, we extracted depression status where the patient was asked if he/she was depressed or anxious, and adherence to MDR-TB treatment status during follow-up based on the proportion of days covered (PDC). For each patient, we calculated a PDC by dividing the number of days covered by MDR-TB treatment delivered over 1 month by 31. Then, we considered a maximum value of PDC during follow-up for each patient as a marker of adherence to MDR-TB treatment. Conventionally, the PDC was dichotomized between good adherence if the PDC was 0.8 or more and poor adherence otherwise (9). Information was collected using a case report form from the MDR-TB registry.

### Statistical analysis

Descriptive statistics (frequencies and percentage or mean and standard deviation [SD]) were used to describe demographic and clinical characteristics of participants at baseline. BMI at baseline was compared between treatment outcome (cured vs died) using Student’s t-test. To analyze the change of BMI over time, we applied the linear mixed (LM) model [9] with correlated auto-regressive intercept and slope random effects to account for inter-patient variability. Time from MDR-TB initiation in months was used as the time scale. Linear, quadratic and cubic effects of time were tested, and then the model that best fitted the data was chosen via the likelihood test ratio. To identify factors that affected the BMI progression, we tested the interaction between the time variable and the given factors. Predictors with a *p*-value less than 0.10 in the univariate analysis, including the interaction terms, were entered into a multivariate regression. Independent predictors that influenced the BMI progression rate were selected through a backward procedure based on the lowest Akaike information criterion.

To identify groups of participants exhibiting different trajectories of BMI, a latent class mixed (LCM) model that accounts for individual and latent group structure variability through random-effects was used [10]. The same fixed and random effects included in the LM model were used for the LCM model. Independent predictors that affected the BMI trajectory from the LM model were used to explain group membership. The best-fitting model with the optimal number of latent classes was selected using the compromise between the lower integrated classification likelihood with the Bayesian information criterion (ICL-BIC), that is the sum of BIC and twice the estimated entropy, and the mean posterior probabilities belonging to each latent class above 0.70 [11, 12]. The ICL-BIC criterion has the advantage of considering the quality of the classification (entropy) in addition to the goodness-of-fit when selecting the optimal number of latent classes. For each patient, the probability of belonging to each trajectory was determined: each patient was then assigned to the group for which her/his probability of belonging to a trajectory was the highest. Distributions of the baseline factors across these classes were compared a posteriori using a chi-squared test for the categorical variables and Student’s t-test for the continuous variables. To assess whether the time to initial sputum smear and culture conversion was directly related to these classes, the Kaplan-Meier method was used and differences in survival time were compared with a log-rank test. All data analyses were done in R (version 3.5.1). Significance was defined as a *p*-value less than 0.05, and all tests were two-sided.

## Results

### Clinical characteristics at baseline

Of 232 patients with MDR-TB, a total of 165 who met the inclusion criterion were analyzed. These patients had a total of 1387 visits, with a median of 5 visits (interquartile range, 3–8 visits). The patients had a mean age of 34.0 (SD 11.3) years, most were male (67%) and lived in urban areas (74%), and their mean BMI was 17.5 (SD 2.7) kg/m^2^. Most patients presented cough (94%), 42 (26%) were HIV-positive, and 13 (9%) had cavities on an initial chest X-ray. According to the history of TB, 134 (81%) patients were previously treated, and 88 and 83% had a positive baseline sputum smear and culture, respectively. 47 patients who had a negative sputum smear and culture at baseline were excluded from the analysis of the association between BMI increase and sputum smear and culture conversion. Therefore, at the study end, 92% (108/118) and 89% (105/118) of patients converted their sputum smear and culture in a median of 59 days (interquartile range: 31–61 days), respectively. Overall, 81% (133/165) of patients were cured by the MDR-TB treatment, and 19% (32/165) died (any reason). Table 1 details the study population according to treatment outcome.

**Table 1:** Population characteristics at baseline

	MDR-TB treatment outcomes		
	Total (***n*** = 165)	Cured (***n*** = 133)	Dead (***n*** = 32)
Age at baseline	33.3 (11.6)	32.2 (10.5)	37.5 (14.7)
Sex (male)	111 (67.3)	97 (72.9)	14 (43.8)
Residence (urban)	122 (73.9)	37 (27.8)	6 (18.8)
BMI	17.5 (2.7)	17.8 (2.6)	16. 0 (2.7)
Initial sputum smear (positive)	145 (87.9)	120 (90.2)	25 (78.1)
Initial sputum culture (positive)	137 (83.0)	113 (88.9)	24 (85.7)
HIV status (positive)	42 (25.6)	27 (20.5)	15 (46.9)
Lung cavities on X-ray (yes)	13 (9.1)	9 (7.7)	4 (15.4)
History of TB treatment (previously treated)	134 (81.2)	114 (85.7)	20 (62.5)
Treatment adherence (yes)	144 (87.3)	128 (96.2)	16 (50.0)
Depression (yes)	8 (4.9)	2 (1.50)	6 (18.8)
Chest pain (yes)	73 (44.2)	54 (40.6)	19 (59.4)
Cough (yes)	156 (94.6)	127 (95.5)	29 (90.6)
Dyspnea (yes)	52 (31.5)	34 (25.6)	18 (56.3)
Nausea (yes)	13 (7.9)	7 (5.3)	6 (18.8)
Vomiting (yes)	17 (10.3)	10 (7.5)	7 (21.9)
Hemoglobin count	10.7 (2.2)	10.8 (2.1)	10.0 (2.6)
Platelet count	388.0 (159.9)	396.1 (152.4)	353.8 (187.1)
Lymphocyte count	1.9 (1.4)	1.8 (0.9)	2.0 (2.6)
Neutrophil count	5.0 (2.6)	5.0 (2.6)	5.5 (2.7)
Creatinine	77.7 (19.6)	76.8 (19.5)	81.3 (19.7)
SGOT	29.6 (5.4)	29.2 (3.8)	31.2 (9.3)
SGPT	32.2 (5.0)	32.2 (4.2)	32.3 (7.6)

Data are expressed as *n* number (%) for categorical variable, and mean (SD); *SD* standard deviation for continuous variable

### Rates of BMI change

**Fig. 2:**
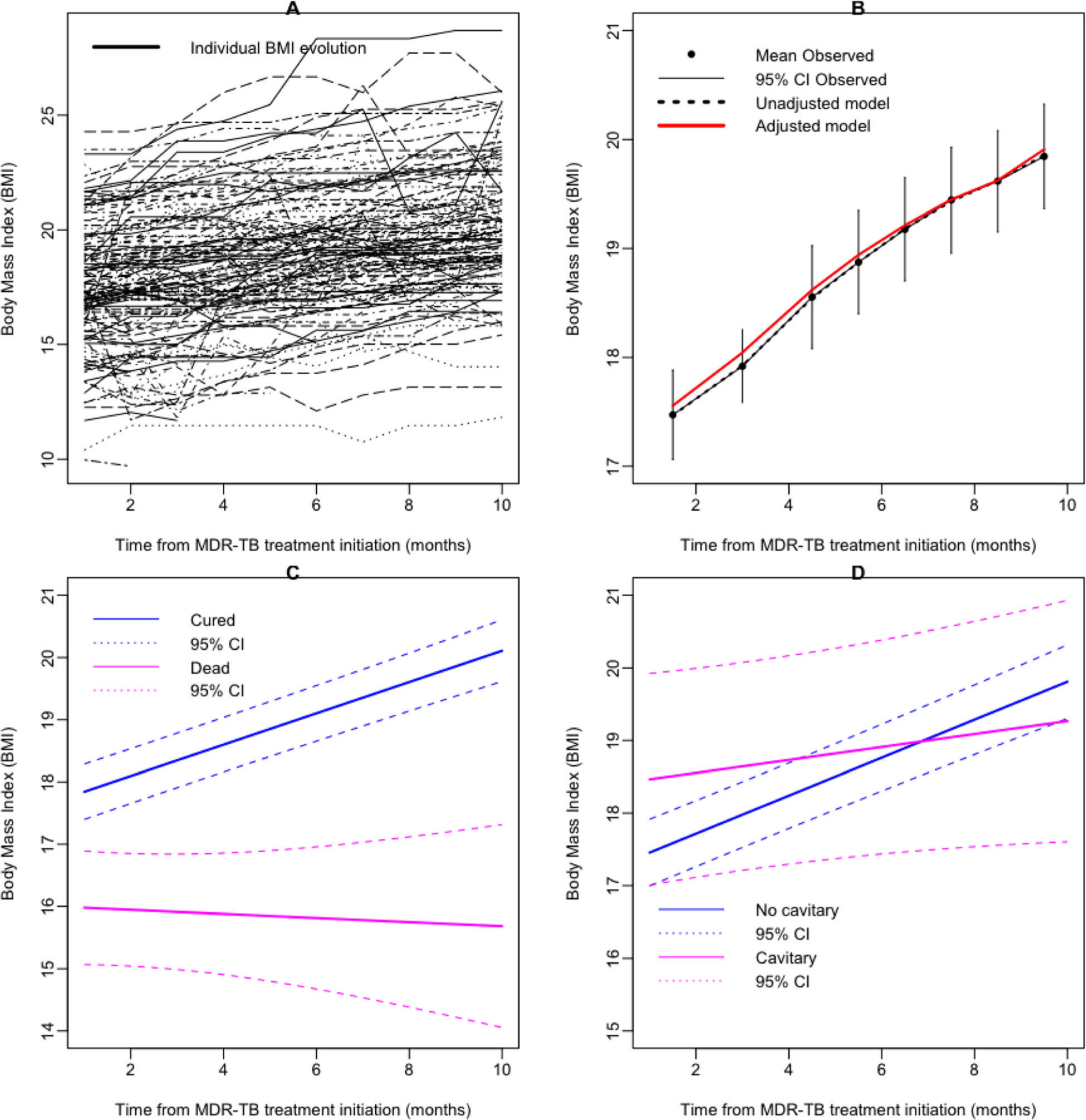
**a** Individual MDR-TB patients’ evolution in body mass index (BMI) (black lines), **b** mean increase in BMI (black dots) and 95% CI (black line), unadjusted prediction from linear mixed model (LMM) (dashed line) and adjusted for treatment outcome and lung cavities on X-ray prediction from LMM (red line); **c** Prediction of BMI progression according to the MDR-TB treatment outcome (solid lines): cured (blue line), dead (magenta line), dashed lines were the 95% CI of the prediction from LMM; **d** Prediction of BMI progression according to the lung cavities on X-ray (solid lines): absence of lung cavities (blue line), presence of lung cavities (magenta line), dashed lines were the 95% CI of the prediction from LMM

Individual BMI trajectories are shown in Fig. 2a. The data on BMI increase were best fitted with a linear model (Fig. 2 b). Overall, the monthly increase in BMI was 0.24 (SE 0.02) per kg/m^2^. Table 1 shows factors associated with the increase in BMI over time in univariate analysis. To identify baseline factors that were independently associated with faster increase of BMI, we applied multivariable modeling. Cure by the MDR-TB treatment (0.24 [SE 0.09] per kg/m^2^; *p* = 0.0205) and the absence of lung cavities on X-ray (0.18 [0.06] per kg/m^2^; *p* = 0.0068) were associated with faster increase in BMI (Table 2). The corresponding predictions and their 95% confidence interval (CI) are displayed in Fig. 2 (c and d). While the increase in BMI was faster in patients reported as cured, BMI decreased in those who died (Fig. 2c).

**Table 2:** Predictors of BMI increase in patients with MDR-TB, multivariate linear mixed random-effect regression

Effects					Estimate	SE	p-value
Intercept	Mean BMI				15.79883	0.54076	**0.00000**
Treatment success (yes)					1.73193	0.58372	**0.00301**
Lung cavities on X-ray (yes)					1.47195	0.76554	0.05451
Slope	Time				0.04917	0.09200	0.59303
Interaction time - Treatment success (yes)					0.21736	0.09380	**0.02049**
Interaction time - Lung cavities on X-ray (yes)					−0.18032	0.06664	**0.00682**

Data are given as mean (SE); *SE* standard error; bold *p* values were < 0.05, which means the corresponding factor was significantly associated with the BMI increase. In the linear mixed model, the repeated measurement of BMI was the dependent variable, and the treatment success and lung cavities on X-ray were the independent variables. Intercept corresponds to the average BMI increase (mean BMI in the table) for patients without lung cavities on X-ray and for the patients who died. Mean BMI was 15.79 kg/m^2^ at baseline. BMI was higher for cured patients [17.52 kg/m^2^ (15.79 + 1.73)] and for patients with lung cavities on X-ray [17.26 kg/m^2^ (15.79 + 1.47)]. Time corresponds to the speed of BMI increase at each visit, which was 0.05 kg/m^2^ for patients who died and for patients without lung cavities on X-ray. The influence of these factors was evaluated by interaction with the given factor and time. The interaction time and treatment line indicate that the BMI increased faster for cured patients [0.27 (0.05 + 0.22) BMI points per month], and the last line in Table 2 means that the BMI increase was slower for patients who had lung cavities on X-ray [− 0.13 (0.05–0.18) BMI points per month]

### Trajectories of BMI and their relationship to baseline factors and sputum (smear and culture) conversion

**Fig. 3:**
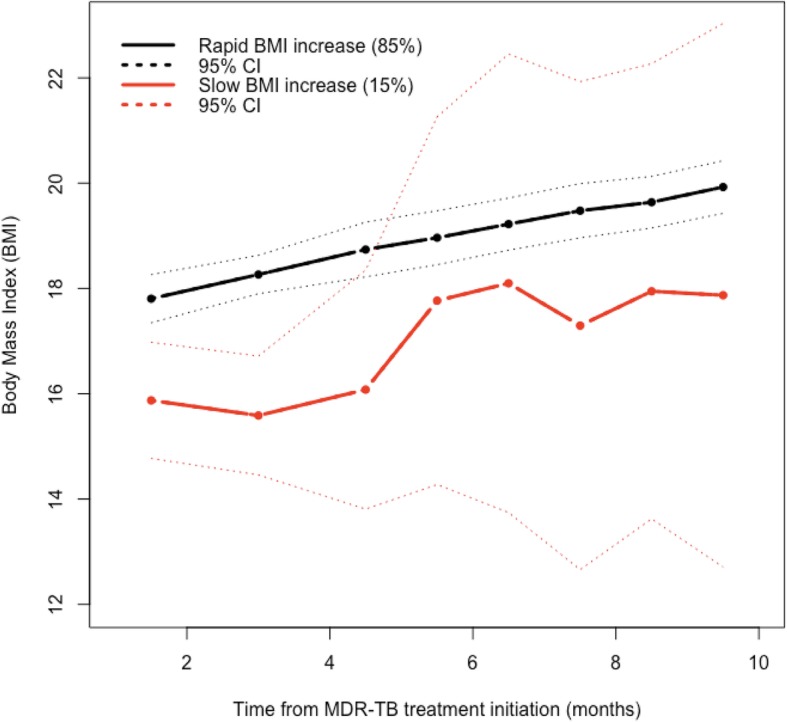
BMI group trajectories in patients with MDR-TB

To identify different trajectories of BMI, we used the LCM model with several latent classes, ranging from 1 to 4 (Additional file 1). Membership of these classes was explained by the treatment outcome and the lung cavities on X-ray. The model with the optimal number of classes selected by the compromise criterion included two different BMI trajectories (Fig. 3). Class 1 (*n* = 121, 85%) was characterized by faster BMI increase over time, named as “Rapid BMI increase”, and Class 2 (*n* = 22, 15%) corresponded to patients who had slow BMI increase over time, named as “Slow BMI increase”. According to the posterior classification provided in Table 3, the patients in the “Slow BMI increase” group were more severely affected at baseline. These patients were mostly female (68%), had no history of TB treatment (41%), a positive HIV infection (59%), and a higher frequency of symptoms including depression (18%), dyspnea (68%), nausea (23%), poor adherence to MDR-TB treatment (64%), lower platelet count (377.7 [181.2]), and higher SGOT (32.7 [9.5]). Using the multivariate logistic regression, the independent factors that were associated with the slow BMI increase were positive HIV infection, non-adherence to MDR-TB treatment, and higher than normal SGOT.

**Table 3:** Description of the posterior classification from the BMI latent class model according to the baseline characteristics for MDR-TB patients

	Slow BMI increase	Rapid BMI increase	
**Characteristics**	**(*n*** = 22)*	**(*n*** = 121)*	***p*-value**
Age at baseline	37.6 (13.8)	32.6 (10.7)	0.1002
Sex (female)	15 (68.2)	33 (27.3)	**0.0004**
BMI (kg/m^2^)	15.8 (2.5)	17.9 (2.5)	0.9989
Initial sputum smear (positive)	19 (86.4)	108 (89.3)	0.2471
Initial sputum culture (positive)	15 (79.0)	104 (89.7)	0.1156
HIV status (positive)	13 (59.1)	22 (18.3)	**0.0001**
History of TB treatment (new case)	9 (40.9)	18 (14.9)	**0.0061**
Treatment adherence (no)	14 (63.6)	4 (3.3)	**<.0001**
Depression (yes)	4 (18.2)	1 (0.83)	**0.0002**
Chest pain (yes)	14 (63.6)	55 (45.5)	0.0550
Cough (yes)	20 (90.9)	120 (99.2)	0.0586
Dyspnea (yes)	15 (68.2)	34 (28.1)	**0.0004**
Nausea (yes)	5 (22.7)	7 (5.79)	**0.0175**
Hemoglobin count	9.8 (2.4)	10.8 (2.1)	0.3888
Platelet count	377.7 (181.2)	384.2 (133.5)	**0.0474**
Lymphocyte count	1.7 (1.6)	1.9 (1.4)	0.3289
Neutrophil count	5.1 (3.0)	4.9 (2.6)	0.3083
Creatinine count	79.5 (17.7)	76.5 (20.2)	0.4928
SGOT	32.7 (9.5)	29.2 (4.1)	**<.0001**
SGPT	34.2 (6.3)	32.2 (4.7)	0.0657

Data are shown as the mean (SD) or number (%); *SD* standard deviation; bold *p* values were < 0.05, which means the corresponding factor was significantly associated with the BMI group latent class. Group membership was explained by treatment outcome and lung cavities on X-ray. *Lung cavities on X-ray at baseline was missing for 22 patients. Distributions of the baseline factors across these classes were compared a posteriori using a chi-squared test for the categorical variables and Student’s t-test for the continuous variables

**Fig. 4:**
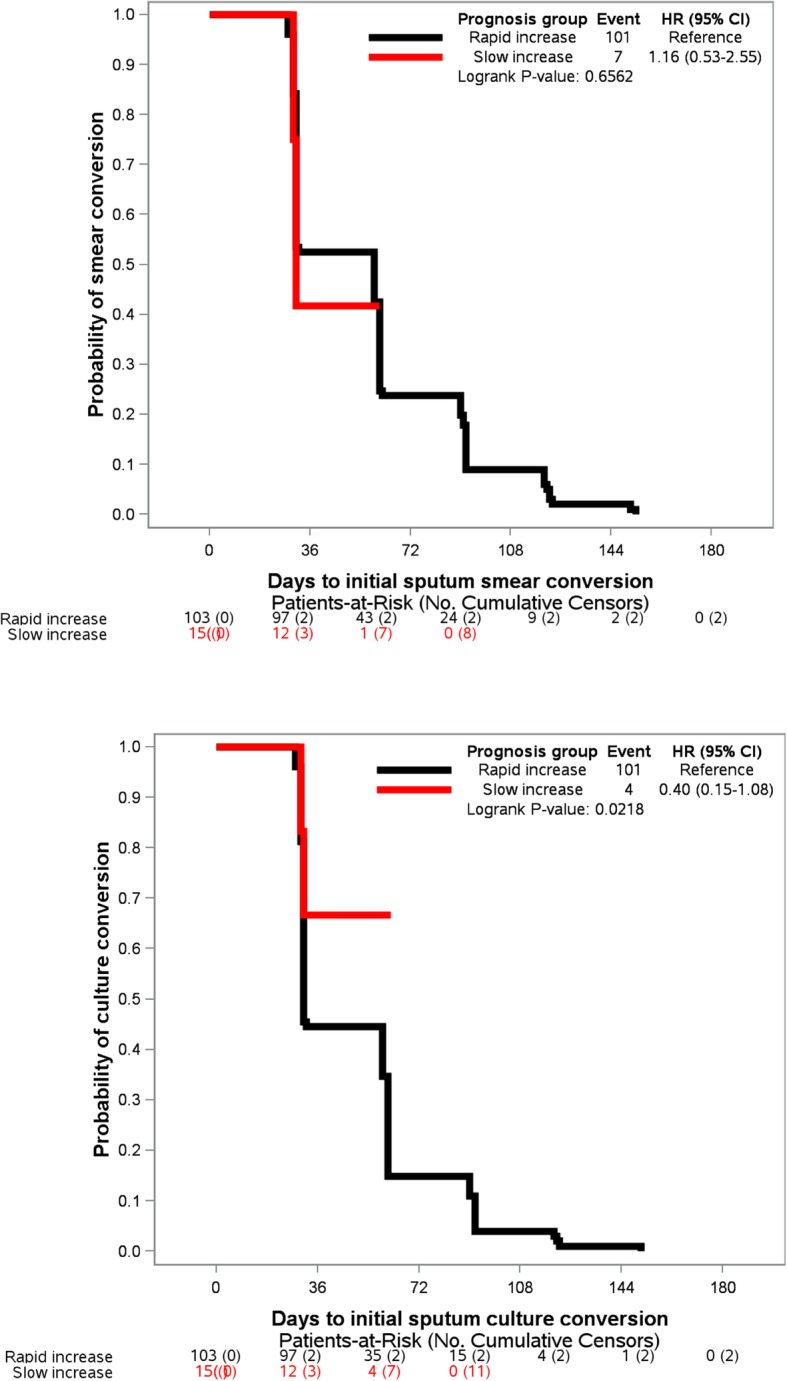
Time-to sputum smear and culture conversions according to the characterization groups from BMI latent classes increase

Patients in the Slow BMI increase group also had a longer time to initial culture conversion (Fig. 4b, log-rank test: *p* = 0.0218), while their time-to-initial sputum smear conversion was comparable to those patients in the rapid BMI increase group (Fig. 4a, *p* = 0.6562).

## Discussion

To the best of our knowledge, this is the first study to identify groups of weight change and to determine factors associated with these groups. Furthermore, these data also suggest that the management of HIV infection and depression status, as well as more therapeutic education to improve treatment adherence may reduce the risk of community transmission from patients with MDR-TB. In addition, the results provide more information to help with patient selection and stratification for the design of future interventional clinical trials.

The mechanism underlying weight loss in patients with MDR-TB is well known [13]. Poverty-induced malnutrition is one of the main causes of weight loss in countries with a high prevalence of TB, such as Guinea. By decreasing the concentration of immunoglobulins, interleukin-2 receptor, and T-cell subset (helper, suppressor-cytotoxic, and natural killer cells), malnutrition further alters the immunity of patients with TB, making them vulnerable to infections such as HIV, and prone to severe clinical presentation and a higher proportion of positive sputum cultures [14]. In addition, socioeconomic status, including the number of household contacts, may increase the risk of the MDR-TB infection. The report of a study conducted in Guinea between 1 January 2017 and 30 September 2018 showed that of 4255 people who underwent the GeneXpert MDR/RIF test, 339 (8%) were identified as household contacts, and 105 (31%) of them were positive for TB (17 MDR-TB and 88 TB sensitive) (data not shown). This prevalence is probably underestimated because only the symptomatic household contacts are depicted. A similar result was reported in China where the positive rate of household contacts was 28% [15]. Furthermore, others risk factors for MDR-TB were reported; they were social determinants of health (monthly low income of the family [< 100 €], stigma, unemployment, prison homelessness, alcoholism and substance abuse), health system weakness (poor organization of TB program, absence or inappropriate clinical guidelines), mental health factors (subjective feeling of sadness, use of sedatives), and clinical factors (history of prior TB treatment, HIV infection, chronic obstructive pulmonary, lung cavitation, and larger burden of bacilli on sputum microscopy) [16–18].

Two profiles of BMI increase were identified: rapid and slow, with the average probability of belonging to the two LCM models being higher, ranging from 0.82 to 0.99, suggesting unambiguous classification (appendix). From a LM model, we found that the BMI increase over time differed according to the treatment outcome. After controlling for lung cavities on X-ray, patients who were cured had gained on average 2.62 kg/m^2^ in BMI at the end of treatment. A previous study reported that patients who were cured had gained on average 3.9 kg at the end of the sixth month [7]. Unlike the LM model, which shows an average gain BMI over time, our analysis showed that the speed of this weight gain was not identical for all patients. The most interesting finding was that the patients in the slow BMI increase group had a poor response to the MDR-TB treatment, suggesting that weight may serve as a potential biomarker to monitor treatment outcome. These patients were characterized by a positive HIV infection, depression symptoms, poor adherence to the MDR-TB treatment, and delay to the culture conversion. This is a relevant finding in public health, particularly in resource-limited settings because it allows better targeting of patients with a high risk of treatment failure and hence better channeling of the resources needed to improve treatment success rates. Strategies such as close monitoring of these patients, therapeutic education to improve treatment adherence, and the setting up of psychiatric consultations to manage depression will help improve the prognosis of these patients and increase their chance of success.

Furthermore, in patients with slow BMI increase, the likelihood of culture conversion was reduced by 65% (HR = 0.35, 95% CI [0.13–0.96]; *p* = 0.0087). This finding was higher than those reported from studies evaluating the impact of baseline weight loss (BMI < 18.5 kg/m^2^) and delay in culture conversion [4, 5]. The reduced chances of culture conversion were 43 and 45% for Indonesian and South Korean patients, respectively. In addition, 89% of our patients showed culture conversion in a median of 2 months, which was higher than the rates of culture conversion reported in Indonesia (80%) [5] and South Korea (70%) [4], suggesting that our MDR-TB treatment program performed reasonably well.

Recently, the superiority of culture conversion over smear conversion in predicting MDR-TB treatment outcomes was demonstrated, with an optimum time point between four and 6 months after treatment commencement. This conclusion supports the WHO recommendation to add culture examination to the sputum smear for the monitoring of MDR-TB patients for better prediction of successful treatment outcomes [19]. Nevertheless, in resource-constrained settings, the sputum culture is resource-intensive, takes time to obtain, is costly, and requires specialized laboratories, equipment and trained staff. We found that in patients with MDR-TB, a stable or decreased weight between two visits is probably a sign of a poor response to treatment, especially in an HIV-infected, depressed woman with lung cavities on X-ray whose treatment adherence was poor. Since the measurement of body weight is easy, rapid, inexpensive, and accessible everywhere, the association between a faster increase in BMI and shorter time to initial culture conversion suggests that weight measurement is a useful surrogate of culture conversion in predicting an early MDR-TB treatment response.

Our study has a number of strengths. First, patients from three referral centers for MDR-TB management in Guinea were evaluated, which reduces selection bias and increases the validity of the extrapolation of our findings to the entire population of Guinean patients with MDR-TB. Second, unlike the conventional mixed linear model used to describe the change in weight over time, our analysis identified a group of patients with poor prognosis (slow BMI increase) as well as the characteristics of these patients. Third, we used a compromise criterion to select the best BMI change groups instead of using only the Bayesian information criterion. As mentioned above, the model with two classes has a higher average posterior probability of up 0.80, suggesting unambiguous classification. Fourth, to account for informative dropout, we applied a sensitivity analysis using a joint model for longitudinal and time to dropout [20]. The results obtained from this joint model were similar to estimations using the standard LM model, suggesting an absence of bias in parameter estimations (data not shown). However, the limits of our study were its retrospective design and small sample size, which had an impact on the internal validity of the study, some missing factors such as diabetes status and other metabolic factors, smoking and alcohol use, information on second-line drug susceptibility, and other anthropometric measurements, such as skin-fold thickness and waist circumference, which could possibly serve as a proxy for weight assessment. Further prospective cohort studies with patient numbers are needed to confirm our findings.

## Conclusion

Our data allowed us to identify two different groups of BMI increase and provided evidence of an association between weight gain and treatment outcome. These findings suggest that monitoring of body weight is potentially a useful surrogate of sputum culture conversion in predicting successful MDR-TB treatment outcome, because patients who showed a rapid BMI increase were more likely to reach culture conversion.

## Supplementary information

**Additional file 1: Appendix: Table 1.** Predictors of BMI increase in patients with MDR-TB, univariate linear mixed random-effect regression. Data are given as mean (SE); SE = standard error. **Table 2.** Summary of the estimated latent class mixed model for the MDR-TB patient data (*n* = 165). Number of latent classes (G) that correspond to the number of fitted models, log-likelihood (L), number of parameters (P), Bayesian information criterion (BIC), integrated classification likelihood with BIC (ICL-BIC), proportion of patients in each latent class (%). Group membership was explained by treatment outcome and lung cavities on X-ray. The model with the lower ICL-BIC was chosen as the best one, which considers the quality of the classification in addition to the goodness of fit when selecting the optimal number of latent classes. **Table 3.** Final posterior classification Number (%) of patients in each class, and mean posterior probabilities of the latent class membership according to the final posterior classification. For example, patients in the rapid BMI increase group were assigned to this class with a mean probability of 99.74% vs a probability of 0.26% of belonging to the slow BMI increase group. Conversely, patients classified in the slow BMI increase group were assigned to this class with a mean probability of 81.9% vs. a probability of 18.1% of belonging to the rapid BMI increase group.

## Data Availability

The data are available upon request (diallodjelo@yahoo.fr).

## Abbreviations

BMI: Body mass index
CI: Confidence interval
HIV: Human immunodeficiency virus
INH: Isoniazid
ICL-BIC: Integrated classification likelihood with Bayesian information criterion
LMM: Linear mixed model
LCMM: Latent class mixed model
MDR-TB: Multidrug-resistant tuberculosis
PV: Predictive value
HR: Hazard ratio
SD: Standard deviation
SE: Standard error
TB: Tuberculosis
WHO: World Health Organization

## References

[1] World Health Organization: Global tuberculosis report 2017. Geneva, Swizerland: WHO press, 2017.

[2] National Tuberculosis Control Program Guinea: Annual report of TB control activity, 2018.

[3] Podewils LJ, Holtz T, Riekstina V, Skripconoka V, Zarovska E, Kirvelaite G (2011). Impact of malnutrition on clinical presentation, clinical course, and mortality in MDR-TB patients. Epidemiol Infect.

[4] Park H-O, Kim S-H, Moon S-H, Byun J-H, Kim J-W, Lee C-E (2016). Association between body mass index and sputum culture conversion among south Korean patients with multidrug resistant tuberculosis in a tuberculosis referral hospital. Infect Chemother.

[5] Putri FA, Burhan E, Nawas A, Soepandi PZ, Sutoyo DK, Agustin H (2014). Body mass index predictive of sputum culture conversion among MDR-TB patients in Indonesia. Int J Tuberc Lung Dis.

[6] Cegielski P, Gler MT, Guilatco R, Johnson JL, Caoili JC, Ershova J (2013). Weight gain and response to treatment for multidrug-resistant tuberculosis. Am J Trop Med Hyg..

[7] Chung-Delgado K, Revilla-Montag A, Guillén-Bravo S, Bernabe-Ortiz A (2014). Weight variation over time and its relevance among multidrug-resistant tuberculosis patients. Int J Infect Dis.

[8] WHO. Definitions and reporting framework for tuberculosis – 2013 revision (updated 2014). Geneva: Switzerland World Health Organization; 2013.

[9] Hansen RA, Kim MM, Song L, Tu W, Wu J, Murray MD (2009). Adherence: comparison of methods to assess medication adherence and classify nonadherence. Ann Pharmacother.

[10] Verbeke G, Molenberghs G. Linear Mixed Models for Longitudinal Data. Springer; 2009.

[11] Commenges D, Jacqmin-Gadda H. Dynamical biostatistical models. Boca Raton: CRC Press/Taylor & Francis; 2016. 374 p.

[12] Han J, Slate EH, Peña EA (2007). Parametric latent class joint model for a longitudinal biomarker and recurrent events. Stat Med..

[13] Byrd RP Jr, Mehta JB, Roy TM. Malnutrition and pulmonary tuberculosis. Clin Infect Dis. 2002;35:634–5. 10.1086/342314 12173145

[14] Scrimshaw NS, SanGiovanni JP (1997). Synergism of nutrition, infection, and immunity: an overview. Am J Clin Nutr.

[15] Lu P, Ding X, Liu Q, Lu W, Martinez L, Sun J (2018). Mediating effect of repeated tuberculosis exposure on the risk of transmission to household contacts of multidrug-resistant tuberculosis patients. Am J Trop Med Hyg.

[16] Stosic M, Vukovic D, Babic D, Antonijevic G, Foley KL, Vujcic I (2018). Risk factors for multidrug-resistant tuberculosis among tuberculosis patients in Serbia: a case-control study. BMC Public Health.

[17] Günther G, van Leth F, Alexandru S, Altet N, Avsar K, Bang D (2015). Multidrug-Resistant Tuberculosis in Europe, 2010–2011. Emerg Infect Dis.

[18] Obuku EA, Meynell C, Kiboss-Kyeyune J, Blankley S, Atuhairwe C, Nabankema E (2012). Socio-demographic determinants and prevalence of tuberculosis knowledge in three slum populations of Uganda. BMC Public Health.

[19] Alene KA, Viney K, Yi H, McBryde ES, Yang K, Bai L (2018). Comparison of the validity of smear and culture conversion as a prognostic marker of treatment outcome in patients with multidrug-resistant tuberculosis. Hasnain SE, éditeur. PLoS One.

[20] Rizopoulos D. Joint models for longitudinal and time-to-event data: with applications in R. Boca Raton: CRC Press; 2012. 261 p.

