# Supplementary material for: Different profiles of body mass index variation among patients with multidrug-resistant tuberculosis: a retrospective cohort study
Source: BMC Infect Dis. 2020 Apr 28;20:315. doi: 10.1186/s12879-020-05028-0 (PMC7189596; doi:10.1186/s12879-020-05028-0)
Supplement: Supplementary file 1 — Additional file 1: Appendix: Table 1. Predictors of BMI increase in patients with MDR-TB, univariate linear mixed random-effect regression. Data are given as mean (SE); SE = standard error. Table 2. Summary of the estimated latent class mixed model for the MDR-TB patient data (n = 165). Number of latent classes (G) that correspond to the number of fitted models, log-likelihood (L), number of parameters (P), Bayesian information criterion (BIC), integrated classification likelihood with BIC (ICL-BIC), proportion of patients in each latent class (%). Group membership was explained by treatment outcome and lung cavities on X-ray. The model with the lower ICL-BIC was chosen as the best one, which considers the quality of the classification in addition to the goodness of fit when selecting the optimal number of latent classes. Table 3. Final posterior classification Number (%) of patients in each class, and mean posterior probabilities of the latent class membership according to the final posterior classification. For example, patients in the rapid BMI increase group were assigned to this class with a mean probability of 99.74% vs a probability of 0.26% of belonging to the slow BMI increase group. Conversely, patients classified in the slow BMI increase group were assigned to this class with a mean probability of 81.9% vs. a probability of 18.1% of belonging to the rapid BMI increase group. [file 12879_2020_5028_MOESM1_ESM.docx]

**Appendix:**

**Table 1: Predictors of BMI increase in patients with MDR-TB, univariate linear mixed random-effect regression**

**Table 2: Summary of the estimated latent class mixed model on the MDR-TB patients’ data (n=165)**

**Table 3: Final posterior classification**

**Table 1: Predictors of BMI increase in patients with MDR-TB, univariate linear mixed random-effect regression**

| **Predictors** |  | **Effect** |  |  | **Estimate** | **SE** | **p-value** |
| --- | --- | --- | --- | --- | --- | --- | --- |
| Age at baseline | | Slope (Time) | |  | 0.33325 | 0.05360 | 0.00000 |
|  |  | Interaction Time - Age | |  | -0.00277 | 0.00156 | 0.07684 |
| Sex |  | Slope (Time) | |  | 0.25289 | 0.03293 | 0.00000 |
|  |  | Interaction Time - Sex (Men) | | | -0.01486 | 0.03842 | 0.69888 |
| Sputum smear (positive) | | Slope (Time) | |  | 0.21464 | 0.05338 | 0.00006 |
|  |  | Interaction Time - Sputum smear (positive) | | | 0.03153 | 0.05618 | 0.57463 |
| Sputum culture (positive) | | Slope (Time) | |  | 0.19960 | 0.05312 | 0.00017 |
|  |  | Interaction Time - Sputum culture (positive) | | | 0.05213 | 0.05621 | 0.35377 |
| Depression (yes) | | Slope (Time) | |  | 0.24586 | 0.01732 | 0.00000 |
|  |  | Interaction Time - Depression (yes) | | | -0.19625 | 0.13400 | 0.14303 |
| Chest pain (yes) | | Slope (Time) | |  | 0.24248 | 0.02192 | 0.00000 |
|  |  | Interaction Time - Chest pain (yes) | | | 0.00084 | 0.03276 | 0.97957 |
| Treatment success (yes) | | Slope (Time) | |  | -0.03269 | 0.08407 | 0.69740 |
|  |  | Interaction Time - Treatment success (yes) | | | 0.28443 | 0.08589 | **0.00093** |
| BMI (kg/m^2^) |  | Slope (Time) | |  | 0.43863 | 0.12896 | 0.00067 |
|  |  | Interaction Time - BMI | |  | -0.01152 | 0.00718 | 0.10846 |
| Lung Cavities on X-ray (yes) | | Slope (Time) | |  | 0.26154 | 0.01839 | 0.00000 |
|  |  | Interaction Time - Lung Cavities on X-ray (yes) | | | -0.17254 | 0.06565 | **0.00858** |
| HIV status (positive) | | Slope (Time) | |  | 0.25104 | 0.01929 | 0.00000 |
|  |  | Interaction Time - HIV status (positive) | | | -0.04706 | 0.04214 | 0.26409 |
| History of TB treatment (previously treated) | | Slope (Time) | |  | 0.18073 | 0.04442 | 0.00005 |
|  |  | Interaction Time - History of TB treatment (previously treated) | | | 0.07316 | 0.04799 | 0.12740 |
| Treatment adherence (yes) | | Slope (Time) | |  | 0.15993 | 0.07752 | 0.03910 |
|  |  | Interaction Time - Treatment adherence (yes) | | | 0.08608 | 0.07950 | 0.27895 |
| Cough (yes) |  | Slope (Time) | |  | 0.31482 | 0.07872 | 0.00006 |
|  |  | Interaction Time - Cough (yes) | | | -0.07559 | 0.08060 | 0.34834 |
| Dyspnea (yes) | | Slope (Time) | |  | 0.24921 | 0.02000 | 0.00000 |
|  |  | Interaction Time - Dyspnea (yes) | | | -0.02478 | 0.03876 | 0.52270 |
| Nausea (yes) | | Slope (Time) | |  | 0.24237 | 0.01779 | 0.00000 |
|  |  | Interaction Time - Nausea (yes) | | | 0.01284 | 0.07322 | 0.86075 |
| Vomiting (yes) | | Slope (Time) | |  | 0.23309 | 0.01754 | 0.00000 |
|  |  | Interaction Time - Vomiting (yes) | | | 0.12094 | 0.06167 | **0.04984** |
| Hemoglobin count | | Slope (Time) | |  | 0.41997 | 0.08668 | 0.00000 |
|  |  | Interaction Time - Vomiting (yes) | | | -0.01645 | 0.00787 | **0.03662** |
| Platelets count | | Slope (Time) | |  | 0.18145 | 0.04808 | 0.00016 |
|  |  | Interaction Time - Platelets count | | | 0.00015 | 0.00011 | 0.17643 |
| Lymphocytes count | | Slope (Time) | |  | 0.29464 | 0.03152 | 0.00000 |
|  |  | Interaction Time - Lymphocytes count | | | -0.02913 | 0.01423 | **0.04065** |
| Neutrophiles count | | Slope (Time) | |  | 0.19123 | 0.03716 | 0.00000 |
|  |  | Interaction Time - Neutrophiles count | | | 0.00980 | 0.00658 | 0.13642 |
| Creatinine count | | Slope (Time) | |  | 0.35156 | 0.06977 | 0.00000 |
|  |  | Interaction Time - Creatinine count | | | -0.00142 | 0.00088 | 0.10891 |
| Live SGOT count | | Slope (Time) | |  | 0.17574 | 0.11917 | 0.14031 |
|  |  | Interaction Time - Live SGOT count | | | 0.00227 | 0.00405 | 0.57515 |
| Liver SGPT count | | Slope (Time) | |  | 0.34917 | 0.11891 | 0.00332 |
|  |  | Interaction Time - Live SGPT count | | | -0.00333 | 0.00367 | 0.36467 |

**Table 2: Summary of the estimated latent class mixed model on the MDR-TB patients’ data (n=165):**

| **G** | **L** | **P** | **BIC** | **ICL-BIC** | **%class1** | **%class2** | **%class3** | **%class4** |
| --- | --- | --- | --- | --- | --- | --- | --- | --- |
| 1 | -1956.18 | 8 | 3953.20 | - | 100.00 |  |  |  |
| **2** | **-1683.77** | **14** | **3437.02** | **3456.55** | **84.62** | **15.38** |  |  |
| 3 | -1674.14 | 20 | 3447.54 | 3486.57 | 79.02 | 15.38 | 5.59 |  |
| 4 | -1664.68 | 26 | 3458.39 | 3476.50 | 2.09 | 5.59 | 76.92 | 15.38 |

Number of latent classes (G) that correspond to the number of fitted models, log-likelihood (L), number of parameters (P), Bayesian Information Criterion (BIC), Integrated classification likelihood with Bayesian information criterion (ICL-BIC), proportion of patients in each latent class (%). Groups membership were explained by treatment outcome and lung cavities on X-ray. Model with the lower ICL-BIC was chosen as the best one, which considers the quality of the classification in addition to the goodness of fit when selecting the optimal number of latent classes.

**Table 3: Final posterior classification:**

|  |  | **Mean posterior probability** | | |  |
| --- | --- | --- | --- | --- | --- |
| **Final** | **Patients** | **to belong to class** | | |  |
| **Classification** | **(%)** | **Rapid BMI increase** | **Slow BMI increase** | | |
| 1 | 121 (84.62) | **0.9974** | 0.0026 |  | |
| 2 | 22 (15.38) | 0.1808 | **0.8192** |  | |

Number (%) of patients in each class, and mean posterior probabilities of the latent class membership according to the final posterior classification. For example, patients in rapid BMI increase were assigned to this class with a mean probability of 99.74% vs a probability of 0.26% to belong to the slow BMI increase. Conversely, patient classified in slow BMI increase were assigned to this class with a mean probability of 81.9% vs. a probability of 18.1% to belong to the Rapid BMI increase.
